# The clinical efficacy and safety of platelet-rich plasma on frozen shoulder: a systematic review and meta-analysis of randomized controlled trials

**DOI:** 10.1186/s12891-024-07629-1

**Published:** 2024-09-06

**Authors:** Wen-Bin Zhang, Yu-Lin Ma, Fei-Long Lu, Hai-Rui Guo, Hao Song, Yi-Mei Hu

**Affiliations:** 1 Department of Orthopedics, Hospital of Chengdu University of Traditional Chinese Medicine, Chengdu, 610072 Sichuan China; 2 Chengdu University of Traditional Chinese Medicine, Chengdu, 611137 Sichuan China

**Keywords:** Frozen shoulder, Periarthritis of shoulder, Adhesive capsulitis, Platelet-rich plasma, PRP, Meta-analysis, Randomized Controlled Trials

## Abstract

**Objective:**

To systematically review the clinical efficacy (pain, function, quality of life) and safety of platelet-rich plasma (PRP) in the treatment of frozen shoulder through meta-analysis, and provide evidence-based medical evidence for the effectiveness of PRP in the treatment of frozen shoulder.

**Methods:**

A search was conducted on international databases (Pubmed, Web of science, Embase) and Chinese databases (CNKI, Wanfang, VIP) to search the clinical studies on the efficacy of platelet-rich plasma in treating frozen shoulder (adhesive capsulitis/periarthritis/50 shoulder) and their corresponding references published from inception until January 2024. Thoroughly excluded literature not meeting the predetermined inclusion criteria, extracted relevant data from the literature, and input it into RevMan5.4 for meta-analysis.

**Results:**

This study ultimately included 14 RCTs, with a total of 1024 patients. The results showed that PRP has significant advantages compared with control groups in VAS (mean difference (MD) *=-0.38, 95%* confidence interval(*CI*)*(-0.73, -0.03), P* = 0.03), UCLA *(MD = 3.31, 95% CI (1.02,5.60),P* = 0.005), DASH (*MD = -4.94,95% CI (-9.34, -0.53),P* = 0.03), SPADI (SPADI Total: *MD =-16.87, 95% CI (-22.84, -10.91), P* < 0.00001; SPADI Pain: *MD =-5.38, 95% CI (-7.80, -2.97), P* < 0.0001; SPADI Disability: *MD =-11.00, 95% CI (-13.61,-8.39), P* < 0.00001), and the active and passive Range of Motion (active flexion: *MD* = 12.70, 95% CI (7.44, 17.95), *P* < 0.00001; passive flexion: *MD* = 9.47, 95% CI(3.80, 15.14), *P* = 0.001; active extension: *MD* = 3.45, 95% CI(2.39, 4.50), *P* < 0.00001; active abduction: *MD* = 13.54, 95% CI(8.42, 18.67), *P* < 0.00001; passive abduction: *MD* = 14.26, 95% CI (5.97, 22.56), *P* = 0.0008; active internal rotation: *MD* = 5.16, 95% CI (1.84, 8.48), *P* = 0.002; passive internal rotation: *MD* = 3.65, 95% CI(1.15, 6.15), *P* = 0.004; active external rotation: *MD* = 10.50, 95% CI(5.47, 15.53), *P* < 0.0001; passive external rotation: *MD* = 6.00, 95% CI (1.82, 10.19), *P* = 0.005) except passive extension (*MD* = 2.25, 95% CI (-0.77, 5.28), *P* = 0.14). In terms of safety, most studies reported no adverse effects, and only one study reported common complications of joint puncture such as swelling and pain after treatment in both PRP and control groups. Previous studies have shown a risk of osteonecrosis caused by corticosteroids. Therefore, the safety of PRP treatment is more reliable.

**Conclusion:**

The results showed that PRP was more durable and safer than corticosteroids and other control groups in the treatment of frozen shoulder.

**Study Design:**

Systematic review.

**Trial registration:**

PROSPERO CRD42022359444, date of registration: 22-09-2022.

**Supplementary Information:**

The online version contains supplementary material available at 10.1186/s12891-024-07629-1.

## Background

Among orthopedic diseases, frozen shoulder (FS) is one of the most prevalent. The term “fifty shoulder” was coined since it is most prevalent in middle-aged and older women over the age of 50 [1]. The range of motion in the affected shoulder is significantly restricted as a result of the condition, which also causes intense and persistent pain that interferes with sleep and lowers quality of life. The disease’s etiology and pathophysiology are yet unknown [2], and there are no established treatment protocols. The majority of available treatments are palliative and symptomatic [3], such as pain alleviation (oral or topical analgesics, block therapy, etc.) and joint motion restoration(manual release and arthroscopic release of shoulder joint) [4]. There is yet no recognized effective treatment for the pathophysiology of frozen shoulder, which can be time-consuming and the effect is limited [5]. In addition, these treatments have great adverse reactions. Oral painkillers can easily lead to gastrointestinal injury [6], oral or injected corticosteroids have the risk of osteonecrosis [7, 8], and the bursa adhesion is easily relapsed by manual or arthroscopic release [9]. Therefore, in order to achieve therapeutic goals, it is crucial to design a successful novel therapeutic strategy.

Platelet-rich plasma (PRP) is a concentrated plasma product derived from platelets through the centrifugation of whole blood [10]. It is rich in anti-inflammatory factors and growth factors and can stimulate the body to increase their secretion. The growth factors present in PRP can promote cell proliferation, repair, and collagen synthesis. Various studies have demonstrated that PRP can enhance cell vitality, promote the proliferation and migration of tendon stem cells [11–13], induce the proliferation and recruitment of mesenchymal stem cells, and facilitate the repair and reconstruction of muscle and soft tissue [14]. This ultimately improves the function of tissues and organs. PRP also shows promise in the treatment of inflammatory responses [15]. It has been found to inhibit inflammatory pathways, such as IL-1β and NF-κB [16, 17], thereby reducing the expression of inflammatory factors and effectively inhibiting inflammation [18]. Clinical applications of PRP have shown remarkable efficacy, particularly in the treatment of musculoskeletal injuries and inflammation [18–20]. As a shoulder disease that is easily confused with frozen shoulder, meta-analyses generally support the efficacy and safety of PRP in treating rotator cuff injuries, as it effectively reduces pain and improves rotator cuff function [21, 22]. Moreover, numerous clinical and experimental studies have found no evidence of any potential hazardous side effects [23, 24], indicating that PRP is a safe form of autologous therapy that has gained popularity in recent years.

The purpose of this study was to offer evidence for the continued and widespread clinical use of PRP for FS by conducting a thorough analysis and evaluation of the clinical efficacy and safety of PRP in the treatment of FS using meta-analysis. It is hypothesized that the efficacy and safety of PRP in the treatment of FS is better than that of the existing conventional control group.

## Methods

The study was conducted by our pre-registered protocol on PROSPERO and the guidance of the PRISMA statement. The PROSPERO registration number for this study is **CRD42022359444.**

### Inclusion and exclusion criteria

Inclusion criteria: (1) Study type: randomized controlled trial (RCTs); (2) Study population: patients with a diagnosis of frozen shoulder (frozen shoulder/ adhesive capsulitis) who have not undergone surgery. The clinical diagnosis of frozen shoulder was based on Shaffer’s criteria [25]; (3) Intervention: articular injection PRP was compared with other treatments (blank control, corticosteroid, normal saline, arthrolysis, ultrasonic physiotherapy, etc.); (4) Outcome indicators: the following study indicators included at least one or more of the following (visual analog score (VAS) of pain, Range of Motion (ROM), The University of California at Los Angeles shoulder rating scale (UCLA), Shoulder Pain Disability Index (SPADI), Disability of the Arm, Shoulder Hand Questionnaire (DASH), etc.).

Exclusion criteria: (1) Literature with incomplete data for analysis; (2) Full text not available; (3) Duplicate literature; (4) Non-randomized controlled trial (non-RCT).

### Search strategy

Search on PubMed, WOS, Embase, CNKI, Wanfang, VIP by computer. The literature was searched for clinical studies related to the use of PRP in the treatment of frozen shoulder (adhesive capsulitis/periarthritis/fifty shoulder) from the time of database construction until January 2024. There are not any language restrictions. The search strategy uses PubMed and Web of Science(WOS) as an example, as shown in supplementary Appendix 1.

### Literature screening and data extraction

The literature obtained after the search was imported into Endnote and duplicates were first removed using Endnote. Two researchers performed independent screening according to the inclusion criteria and exclusion criteria (Ma and Song), read the titles and abstracts of the de-duplicated literature to initially exclude non-RCT studies, and then acquired and read the full text of the remaining literature. Data information for the included literature was then extracted. The information extracted included basic characteristics of each literature (author’s name, country, year of publication, etc.), basic characteristics of the cases (intervention and control measures, sample size, patient sex ratio, age, follow-up time, etc.), primary and secondary outcomes of the trials, conclusions of the trials, quality assessment methods, etc. Disagreements were assessed by a third investigator and consensus finalization was reached. Once extracted without disagreement, the data were entered into Rev Man 5.4 software for Meta-analysis.

### Quality assessment

The quality of the literature for RCTs was evaluated according to the Cochrane Collaboration’s Randomized Controlled Trial tool [26]. Each RCT study screened for inclusion was assessed according to seven characteristics (random sequence generation, allocation concealment, blinding of participants and personnel, blinding of outcome assessment, incomplete outcome data, selective reporting, and other bias) and was rated as low, unclear or high risk of bias. When each item was rated as ‘low risk’, the study was considered to have an overall low risk of bias, and when 1 or 2 items were categorized as ‘high risk’ or ‘unclear risk’ the study was considered to be at medium risk of bias and the literature was considered to be at high risk of bias if there were > 2 items or > 3 items of “high risk” plus “unclear risk" [27]. Funnel plots were used to analyze whether publication bias existed in the included literature.

### Statistical analysis

Baseline data were subtracted from the data of each follow-up to obtain the change values of all outcome indicators, and then the corresponding change values of each follow-up period were imported into RevMan 5.4 software for meta-analysis of the data. Relative risk (RR) and 95% confidence interval (CI) were selected for dichotomous variables. For the continuous variables, Mean difference (MD) and 95% CI were selected. In terms of heterogeneity, Chi-square test and I² test were selected for evaluation. When I² ≤ 50% and *P* ≥ 0.1, shows the heterogeneity was low, then the fixed effect model should be selected. When the I² > 50% and the *P* < 0.1, shows the heterogeneity is high, if the cause of heterogeneity could not be found, a random effects model was selected for analysis. As the control groups of the included studies differed in their treatment modalities, a random effects model was used for all analyses for a relatively conservative analysis. Due to the different follow-up time nodes of various studies, the follow-up time nodes were distinguished in this study according to the development trend of frozen shoulder disease course, the follow-up time was divided into early follow-up (≤ 4 weeks), mid-term follow-up (4–24 weeks), and late follow-up (≥ 24 weeks), all of the follow-up data of the corresponding periods were included in the analysis. Differences were statistically significant when *P* < 0.05.

## Results

### Literature search results

**Fig. 1:**
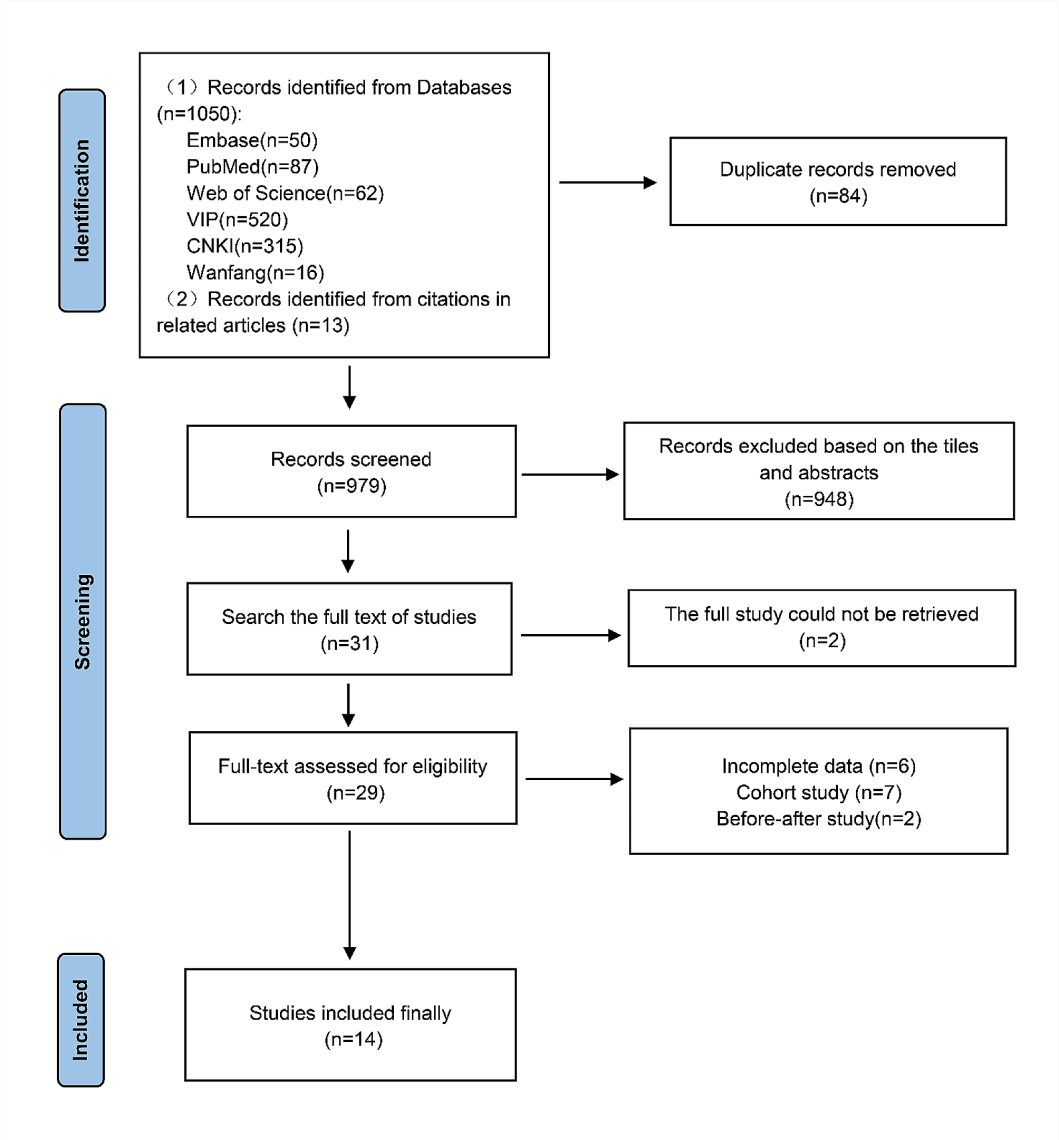
Flow diagram of the selection process

A total of 1063 literatures were retrieved according to the search strategy. After initial screening by title, abstract, the remaining 31 publications, downloaded and carefully read in full, excluded 17 publications (6 with incomplete data [28–33], 2 with unavailable full text [34, 35], 7 cohort studies [36–42] and 2 before-after studies [43, 44] and finally included 14 RCTs^1, 5, 45–56^. A flow chart of the literature screening is presented in Fig. 1.

A total of 1024 patients were enrolled in the studies, of which 515 were treated with PRP, 15 with normal saline injection, 449 with articular injection corticosteroids (AICS), 20 with blank control, and 25 with arthrolysis. The basic characteristics of the included literature is presented in Table 1.

**Table 1:** Basic characteristics of the included literature

First author(Year)	Country	Study design	Sample size	Female(%)	Mean age(years old)	Symptom duration (Month)	Follow-up	Outcome
Lin, J(2018) [1]	China	RCT	PRP:30 CS:30	PRP: 21(70.0) CS: 19(63.3)	PRP: 59.80 ± 4.30 CS: 58.20 ± 4.60	/	6 M	①②
Karabas, C (2021) [47]	Turkey	RCT	PRP:20 N:20	PRP: 6(30.0) N: 13(65.0)	PRP: 57.30 ± 7.30 N: 56.80 ± 5.90	PRP: 5.60 ± 4.00 N: 4.40 ± 3.20	12 W	①③④
Kothari, S(2017) [48]	India	RCT	PRP:62 CS:60 U:58	PRP: 28(45.2) CS: 31(51.7) U: 35(60.3)	PRP: 51.90 ± 10.10 CS: 52.70 ± 8.60 U: 51.20 ± 11.70	PRP: 4.10 ± 2.50 CS: 5.20 ± 2.80 U: 4.70 ± 2.10	12 W	①③⑤
Burcu unlu(2021) [51]	Turkey	RCT	PRP:17 NS:15	PRP: 11(64.7) NS: 10(66.7)	PRP: 53.12 ± 12.25 NS: 57.17 ± 7.19	PRP: 4.65 ± 1.67 NS: 6.00 ± 1.72	3 M	①③④
Jain(2021) [52]	India	RCT	PRP:25 CS:25	/	All: >18	/	3 M	①
Thu (2020) [50]	Korea	RCT	PRP:31 CS:30	PRP: 27(87.1) CS: 21(70.0)	PRP: 52.84 ± 6.92 CS: 57.17 ± 6.93	/	6 W	①③⑤
Lin JunHong (2017) [45]	China	RCT	PRP:30 CS:30	All: 33(55.0)	All: Mean:58.20 Age range: (5-30)	All: 3–18	6 M	①②
Shahzad, H.F.(2021) [49]	America	RCT	PRP:102 CS:100	PRP: 59(57.8) CS: 59(59.0)	PRP: 52.41 ± 4.67 CS: 53.00 ± 3.74	PRP: 5.57 ± 1.49 CS: 4.86 ± 1.50	12 W	①②
Zhang Wei(2021) [53]	China	RCT	PRP:25 A:25	PRP: 16(64.0) A: 11(44.0)	PRP: 53.50 ± 5.41 A: 55.33 ± 4.35	PRP: 6.47 ± 2.01 A: 7.06 ± 2.14	12 M	①③
Iqra Mehak(2022) [46]	India	RCT	PRP:20 CS:20	/	All: >18 Age range: (5-25)	/	6 W	④
Gupta (2022) [54]	India	RCT	PRP:30 CS:30	PRP: 17(56.8) CS: 18(60.0)	PRP: 47.8 ± 9.56 CS: 46.7 ± 7.13	/	6 M	①⑤
Upadhyay (2020) [5]	India	RCT	PRP:60 CS:60	PRP: 35(58.3) CS: 35(58.3)	/	/	6 M	④
Kumar (2021) [55]	India	RCT	PRP:29 CS:30	PRP: 22(73.3) CS: 14(46.7)	Age range: (30–70)	/	6 M	④⑥
Somisetty, TK(2023) [56]	India	RCT	PRP:34 CS:34	PRP: 19(55.9) CS: 14(41.2)	PRP: 58.3 ± 8.1 CS: 58.5 ± 7.7	No statistically significant difference in the proportion of duration (months) between the study groups (*P* = 0.105).	24 W	①④⑤

①:VAS;②:UCLA;③:ROM;④:SPADI;⑤:DASH:⑥:Treatment effect grading

PRP: platelet-rich plasma; N:blank control; CS: corticosteroid; NS: normal saline; A: arthrolysis; U: ultrasonic physiotherapy

### Quality assessment result

**Fig. 2:**
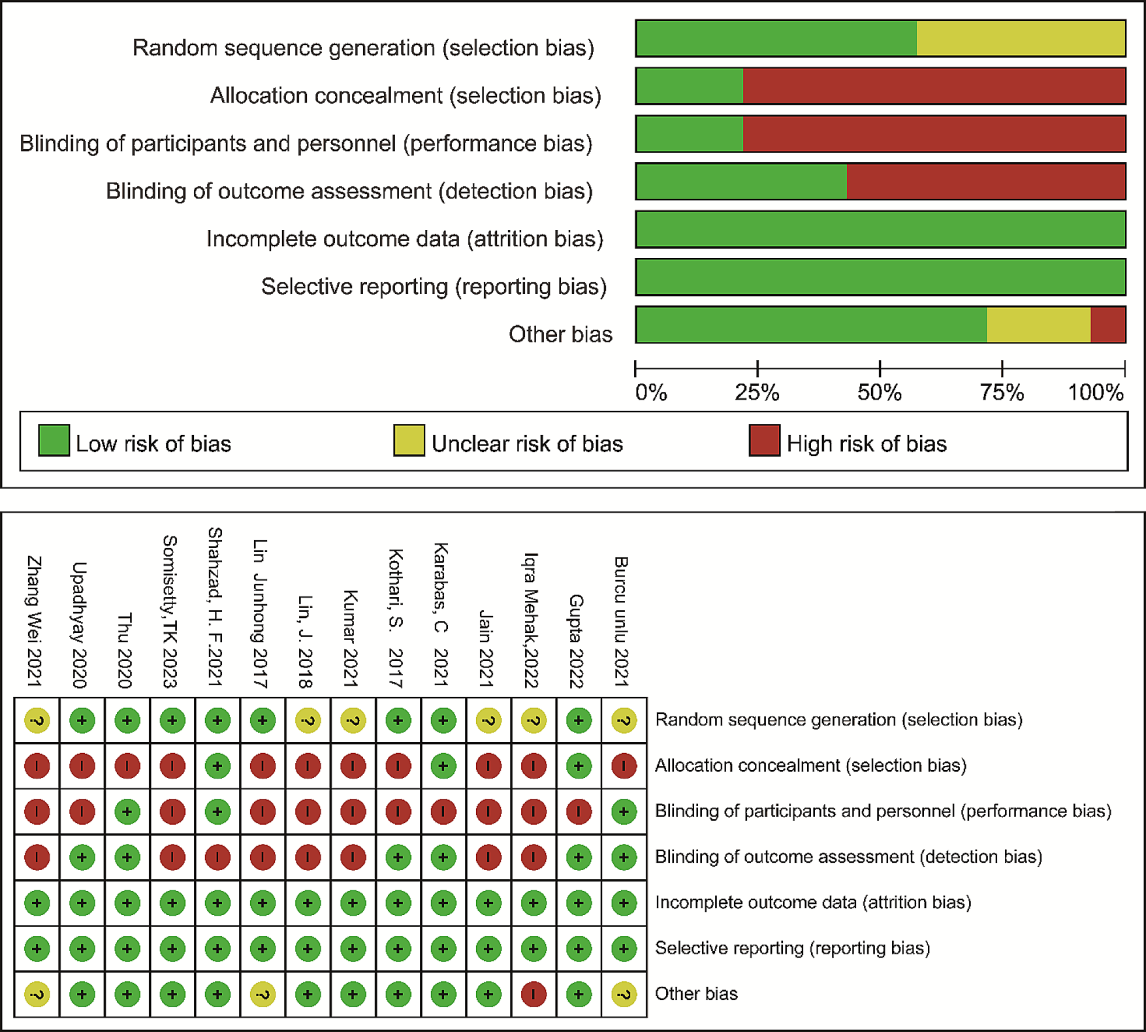
Quality assessment results of the RCT studies

6 of the included studies^1, 46, 51–53, 55^ describe only randomization, without a specific description of the randomization method, and are defined as “unclear risk”; 11 studies^1, 5, 45, 46, 48, 50–53, 55, 56^ do not describe allocation concealment and are defined as “high risk “; for blinding, only 7 studies^5, 47–51, 54^ specified blinded measures, the rest of the literature^1, 45, 46, 52, 53, 55, 56^ defined as “high risk”; for other biases(conflict of interest, limitation, etc.), 3 studies [45, 51, 53] were not described and defined as “unclear risk” and 1 study [46] for which the author was a journal editorial board member, was defined as “high risk”; all outcome indicators were reported in full in the literature, with no selective reporting, refer to Fig. 2.

### Meta-analysis results

The follow-up time was divided into early follow-up (≤ 4 weeks), mid-term follow-up (4–24 weeks), and late follow-up (≥ 24 weeks), all of the follow-up data of the corresponding periods were included in the analysis. In order to evaluate the effect of different control measures and different follow-up time on the analysis results, subgroup analysis was performed for different control measures and different follow-up time.

### Visual analog score of pain (VAS)

**Fig. 3:**
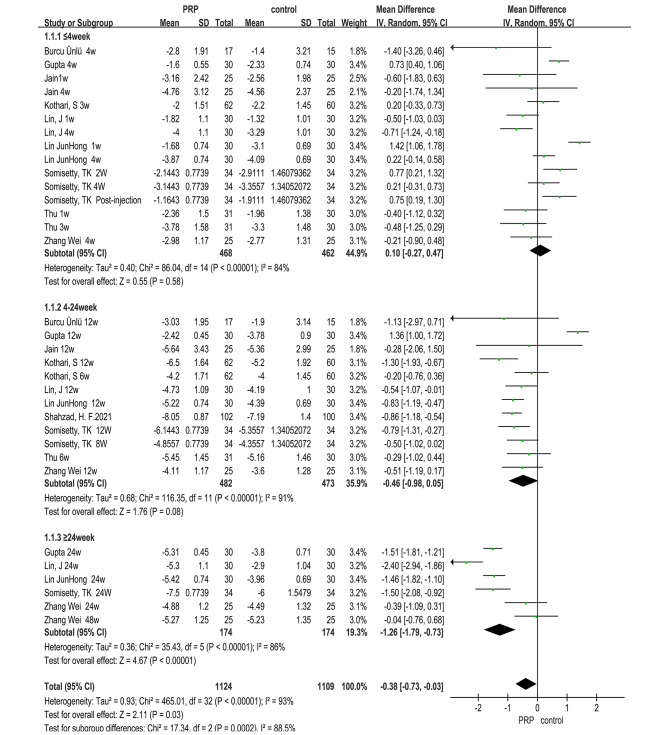
Forest plot for meta-analysis of VAS score

A total of 12 RCTs^1, 32, 45, 47–54, 56^ used VAS scores as an outcome indicator, but one article [47] delivered a VAS score that was appraised differently from the rest of the literature and was not appropriate for the analysis. The fact that PRP is superior to CS in improving VAS was also corroborated by one other study [32]. However, this study was excluded since it only contained graphs and lacked specific data (*Mean ± SD, Median(IQR)*), which prevented meta-analysis. Therefore, VAS scores of the remaining 10 studies were analyzed. Although the effectiveness of the early and intermediate follow-up PRP was not substantially different from that of the control group(≤ 4week: *MD = 0.10, 95% CI(-0.27, 0.47), P* = 0.58; 4-24week: *MD =-0.46, 95% CI (-0.98, 0.05), P* = 0.08), the analysis revealed that the PRP group was significantly better than the control group in VAS improvement at the late stage (≥ 24 weeks: *MD =-1.26, 95% CI(-1.79, -0.73), P*<0.00001), and that the PRP group was also significantly superior than the control group in VAS improvement overall(*MD =-0.38, 95% CI (-0.73, -0.03), P* = 0.03) (see Fig. 3.).

Subgroup analysis of VAS in the three follow-up periods was conducted according to the differences of the control group. The results showed that although the improvement of VAS in the PRP group was stronger than that in the non-steroid control group during the early and middle follow-up compared with CS, this advantage was not statistically significant(≤ 4week: CS (*MD = 0.17, 95% CI (-0.21, 0.56), P* = 0.38); Others (*MD =-0.48, 95% CI (-1.45, 0.50),P* = 0.34)); (4-24week: CS(*MD =-0.42, 95% CI)-0.99, 0.15), P* = 0.15); Others (*MD =-0.58, 95% CI (-1.22, 0.05), P* = 0.07)). The analysis of late follow-up showed that in terms of VAS improvement, the advantage of the PRP group over the steroid group was more pronounced than that of the non-steroid group, and the pooled results also showed a statistically significant advantage for PRP versus all control groups (≥ 24week: CS (*MD =-1.68, 95% CI (-2.06, -1.31), P*<0.00001); Others (*MD =-0.22, 95% CI(-0.72, 0.28), P* = 0.39); Total(*MD =-1.26, 95% CI(-1.79,-0.73), P*<0.00001)).

According to the overall analysis of follow-up time, it can be seen that with the increase of follow-up time, the therapeutic advantage of PRP has a gradually increasing trend. As is shown in Fig. 3. and Supplemental Table 2.

### Range of motion(ROM)

A total of six RCTs^47–51, 53^ used post-treatment shoulder mobility in all directions as an outcome index to assess the recovery of shoulder function in patients after treatment, including 10 indicators of active and passive activity in 5 directions, such as flexion, extension, abduction, internal rotation and external rotation.

**Fig. 4:**
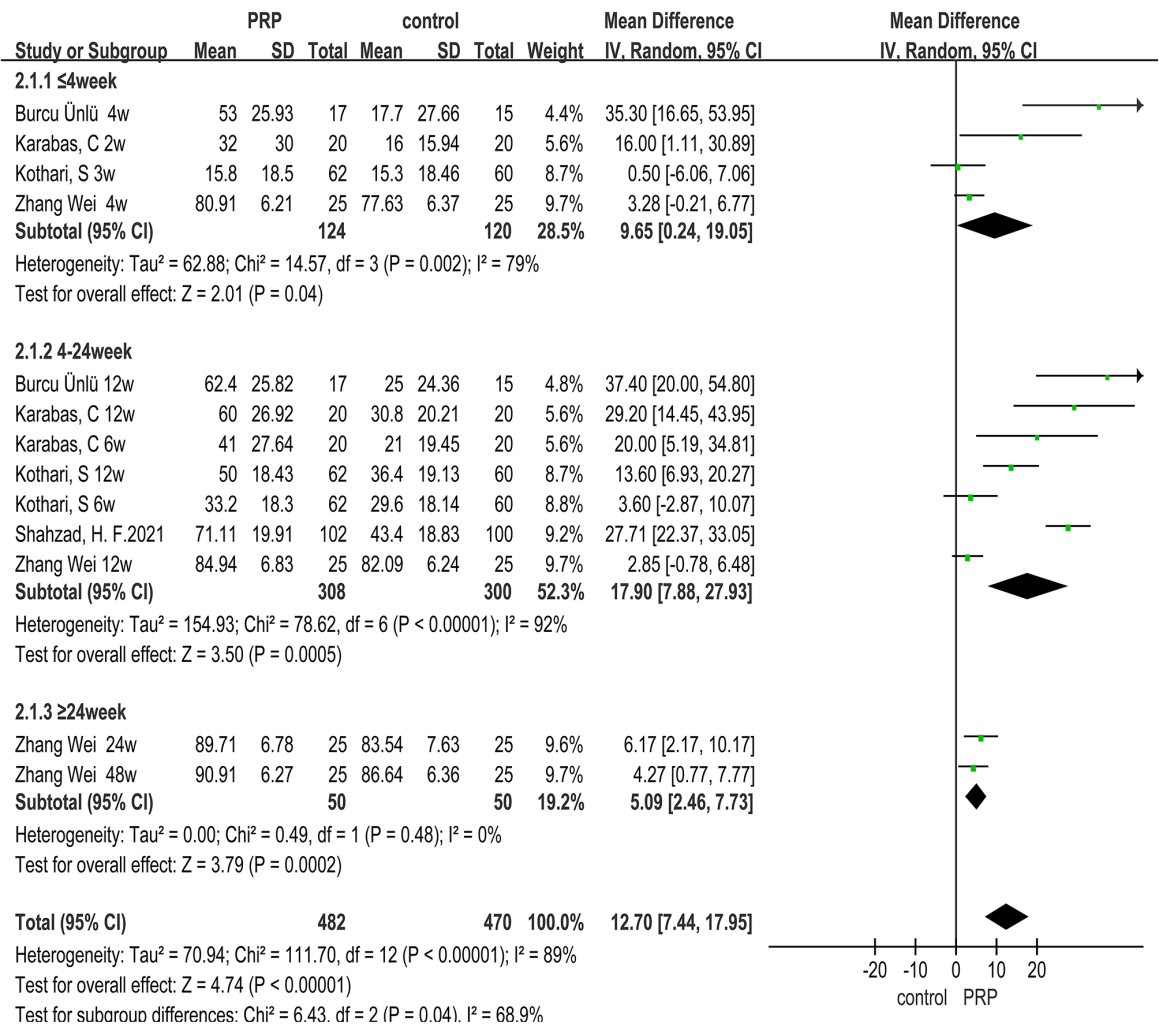
Forest plot for Meta-analysis of active flexion

Analysis of the data showed that, with the exception of passive extension (*MD = 2.25, 95% CI (-0.77, 5.28), P* = 0.14), there was a significant advantage in active and passive shoulder mobility in all directions after treatment in the PRP group compared to the control group (active flexion: *MD = 12.70, 95% CI (7.44, 17.95), P* < 0.00001; passive flexion: *MD = 9.47, 95% CI (3.80, 15.14), P* = 0.001; active extension: *MD = 3.45, 95% CI (2.39, 4.50), P* < 0.00001; active abduction: *MD = 13.54, 95% CI (8.42, 18.67), P* < 0.00001; passive abduction: *MD = 14.26, 95% CI (5.97, 22.56), P* = 0.0008; active internal rotation: *MD = 5.16, 95% CI (1.84, 8.48), P* = 0.002; passive internal rotation: *MD = 3.65, 95% CI (1.15, 6.15), P* = 0.004; active external rotation: *MD = 10.50, 95% CI ( 5.47, 15.53), P* < 0.0001; passive external rotation: *MD = 6.00, 95% CI (1.82, 10.19), P* = 0.005), refer to Fig. 4. for forest plots (with active flexion as an example). The rest of the results is shown in the Supplemental Table 1.

Since there was no non-steroid control group for extension, subgroup analysis of the remaining range of motion data in other directions was performed according to a different control approach for each follow-up period. The results showed that there was no significant difference between the PRP group and the control group in the early stage (≤ 4week: passive flexion (*MD = 5.63, 95% CI (-2.50, 13.77), P* = 0.17); passive abduction *(MD = 9.54, 95% CI (-1.75, 20.82), P* = 0.10); active internal rotation *(MD = 0.91, 95% CI (-3.48, 5.30), P* = 0.68); passive internal rotation *(MD = 1.27, 95% CI (-3.09, 5.62), P* = 0.57); active external rotation *(MD = 4.13, 95% CI ( -1.66, 9.92), P* = 0.16); passive external rotation *(MD = 2.26, 95% CI (-2.12, 6.63), P* = 0.31)), except for a significant advantage in active flexion and active abduction(≤ 4week: active flexion (*MD = 9.65, 95% CI(0.24, 19.05), P* = 0.04); active abduction (*MD = 5.63, 95% CI (-2.50, 13.77), P* = 0.04)). At mid-term and late follow-up, the improvement in range of motion in the PRP group was significantly better than that in the control group in all directions (4-24week: active flexion (*MD = 17.90, 95% CI (7.88, 27.93), P* = 0.0005); passive flexion (*MD = 12.55, 95% CI (4.88, 20.22), P* = 0.001); active abduction (*MD = 19.39, 95% CI (10.47, 28.31), P*<0.0001); passive abduction (*MD = 18.23, 95% CI (5.55, 30.91), P* = 0.005); active internal rotation (*MD = 6.43, 95% CI (2.71, 10.14), P* = 0.0007); passive internal rotation *(MD = 4.80, 95% CI (1.51, 8.09), P* = 0.004); active external rotation (*MD = 12.74, 95% CI ( 7.03, 18.46), P*<0.0001); passive external rotation (*MD = 8.68, 95% CI (2.33, 15.04), P* = 0.007). ≥24week: active flexion (*MD = 5.09, 95% CI (2.46, 7.73), P* = 0.0002); active abduction (*MD = 4.80, 95% CI (2.22, 7.38), P* = 0.0003)). Moreover, in terms of the improvement of range of motion, the advantage of PRP compared with Others was more pronounced at any period than when compared with the CS group. As is shown in Supplemental Table 2.

According to the overall analysis of follow-up time, it can be seen that from the early to the middle follow-up, the therapeutic advantage of PRP has a more obvious trend, but in the late follow-up stage, this advantage has a tendency to weaken. As is shown in Fig. 4. and Supplemental Table 2.

Two other studies [32, 33] reported follow-up results of improvement in ROM, but they both lacked detailed data (*Mean ± SD, Median(IQR)*) for meta-analysis and were not included.

### Shoulder pain disability index (SPADI)

A total of five RCTs^5, 46, 47, 51, 55^ reported on the post-treatment shoulder pain disability index (SPADI), of which two reported only pain and disability (SPADI Pain, SPADI Disability) but not have SPADI Total [46, 55], while the other three reported on all three indices.

**Fig. 5:**
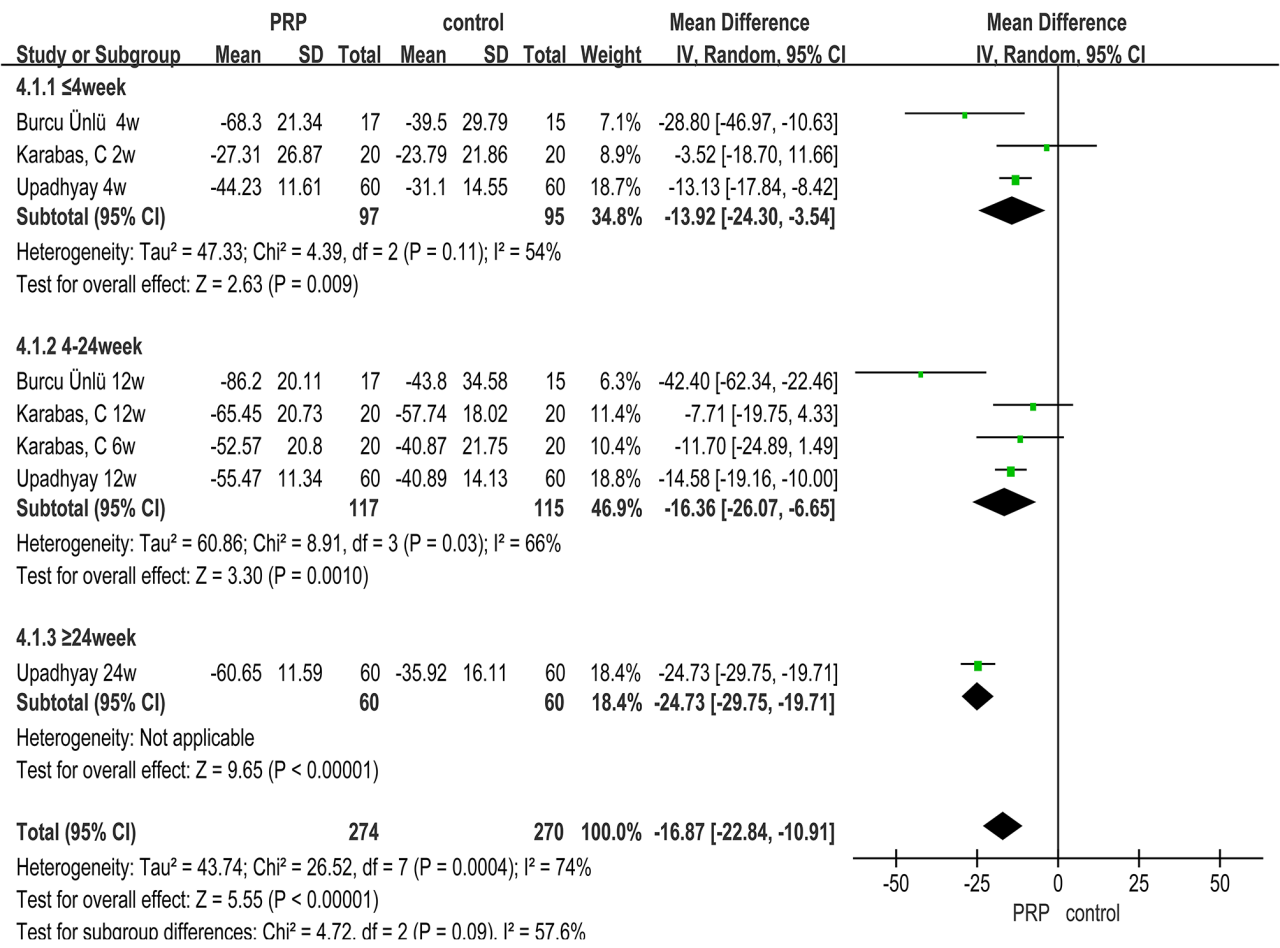
Forest plot for Meta-analysis of SPADI Total

Analysis of the data showed that SPADI improved significantly better in the PRP group than in the control group after treatment. SPADI Total (*MD =-16.87, 95% CI (-22.84, -10.91), P*<0.00001); SPADI Pain (*MD=-5.38, 95% CI (-7.80, -2.97), P* < 0.0001); SPADI Disability (*MD =-11.00, 95% CI (-13.61, -8.39), P* < 0.00001), refer to Fig. 5. for forest plots (with SPADI Total as an example).

Except for SPADI Pain, the other two indicators showed a significant advantage in the PRP group compared with the control group in the early stage (≤ 4week: SPADI Total (*MD =-13.92, 95% CI (*-24.30, -3.54), *P* = 0.009);SPADI Disability (*MD =-7.33, 95% CI (-12.16, -2.49)*, *P* = 0.003)). And from the analysis of different periods, with the increase of follow-up time, the therapeutic advantage of PRP has a more obvious trend. See Fig. 5. and Supplemental Table 1.

According to the different follow-up periods, the subgroup analysis of SPADI was conducted from the perspective of different control methods. The results showed that in the early and middle stages, only SPADI Pain showed a stronger advantage of PRP compared with Others than with CS group. While the other two aspects show that the advantage of PRP over CS group was stronger than that over Others group. There were no other controls in the late stage, so there was no comparison. See Supplemental Table 2.

The fact that PRP is superior to CS in improving SPADI was also corroborated by one other study [56]. However, this study was excluded since it only contained graphs and lacked specific data (*Mean ± SD, Median(IQR)*), which prevented meta-analysis.

### The University of California at Los Angeles shoulder rating scale (UCLA)

A total of three RCTs [1, 45, 49] used the UCLA score as an outcome indicator. The analysis showed that overall the UCLA score was significantly better in the PRP group than in the control group after treatment (*MD = 3.31, 95% CI (1.02, 5.60), P* = 0.005), and according to the analysis results of different follow-up periods, it can be seen that from the early to the middle follow-up, the therapeutic advantage of PRP has a more obvious trend, but in the late follow-up stage, this advantage has a tendency to weaken or even disappear, refer to Supplemental Table 1.

### Disability of the arm, shoulder, and Hand Questionnaire (DASH)

A total of 3 RCTs [48, 50, 54] used the DASH score as an outcome indicator. The meta-analysis results revealed a significant difference in DASH between the PRP group and the control group following treatment (*MD =-4.94, 95% CI (-9.34, -0.53), P* = 0.03). And according to the analysis of different follow-up periods, it can be seen that with the increase of follow-up time, the therapeutic advantage of PRP has a more obvious trend, refer to Supplemental Table 1.

An additional study [56] further supported the finding that PRP was superior to CS in improving DASH. Nevertheless, this particular study was eliminated due to the absence of pertinent data (*Mean ± SD, Median(IQR)*).

### Therapeutic effectiveness

Only 2 RCTs [52, 55] in the included literature utilized treatment outcome grading as an outcome indicator, which in turn allowed conversion to a therapeutic effectiveness rate. The effective rates were 68.0% and 86.2% in PRP group and 52.0% and 10.0% in control group, respectively, the Meta-analysis result is shown in Supplemental Table 1. Another 2 cohort study [37, 38] and a study [30] excluded due to incomplete data also used treatment outcome grading as an outcome indicator, with the effective rates were 97.3%, 87.0% and 92.0% in the PRP group and 81.1%, 84.4% and 81.0% in the control group. Therefore, the results of all three studies showed that the PRP treatment group was more effective compared to the control group, but the advantage wasn’t statistically significant.

### Heterogeneity analysis

Except for a few outcome indicators in ROM, which showed little heterogeneity due to fewer included studies, the heterogeneity test results of all the other outcome indicators showed great heterogeneity: *P* < 0.05, *I*^*2*^ > 50%. Through the leave-one-out analysis, no obvious source of heterogeneity was found, and the reasons for this were that there was no consistent international standard on the use and production of PRP, and the treatment methods of the control group were also different, which may be the sources of heterogeneity. In terms of treatment methods, a total of one study was arthrolysis, one was blank control, one was normal saline control, and the rest were corticosteroid (the types of steroids were also different). For conservative analysis, the random effects model was used for analysis in this study, which has been discussed in the methods section.

### Bias analysis

**Fig. 6:**
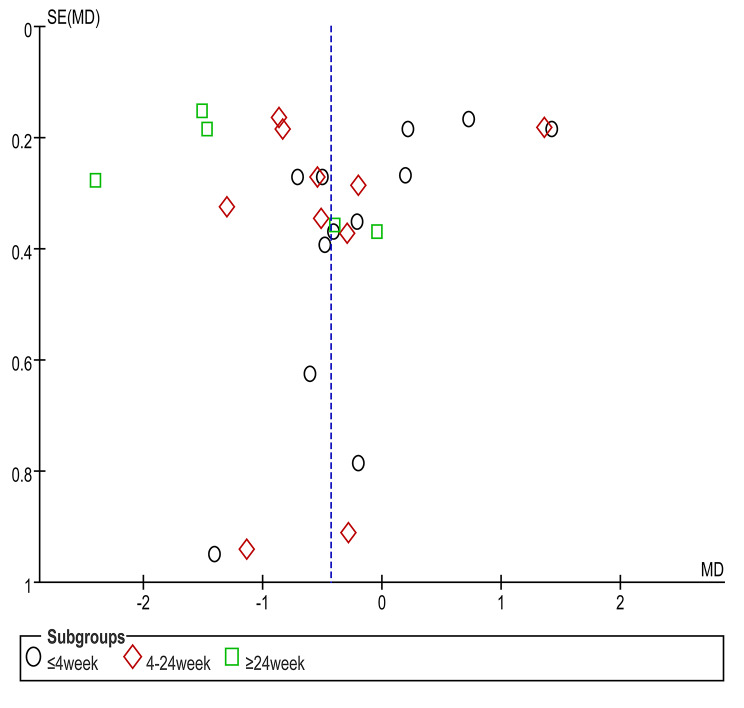
Funnel plot of VAS

The outcome index of VAS score with the largest number of included literatures was analyzed for bias. A symmetrical distribution of funnel plots was observed, with most studies located at the top of the funnel plots, and no significant risk of bias was discovered. The funnel plot is given in Fig. 6.

## Discussion

The analysis of this study showed that PRP reduces shoulder VAS score and relieves pain, progressing over time from an insignificant effect in the early follow-up (≤ 4 weeks), to the most significant effect in the mid-term follow-up (4–24 weeks), and then gradually diminishing or even disappearing in the late follow-up (≥ 24 weeks). This phenomenon is basically consistent with previous studies on PRP [19, 57]. The main reason may be that with the progress of treatment, the inflammation is almost controlled, and the soft tissues such as tendons and bursae are almost repaired, so the effect gradually diminishes or disappears. This trend was also demonstrated in ROM, UCLA, which enhanced shoulder function and alleviated pain. The study findings demonstrate that the use of PRP in treating frozen shoulder is most effective during mid-term follow-up (4–24 weeks), which is consistent with existing research [20] indicating that the optimal period for PRP treatment ranges from 3 to 6 months.

In the analyzed RCTs and excluded cohort studies, the pattern of highest effectiveness during the mid-term follow-up, followed by a decrease or loss of effectiveness during the long-term follow-up, remained predominantly consistent. In addition to the fact that the efficacy of PRP itself diminished or disappeared over time, this result may also be related to the natural course of FS itself. As a self-limiting disease, the progression of FS can be divided into three stages: Stage 1(2–9 months), is characterized by progressively increasing pain and stiffness and is called the freezing stage. 4–12 months is the stage 2, characterized by persistent stiffness and pain, called the frozen stage; 12–42 months is the stage 3, called the thawing period, the pain gradually alleviates, and the joint motion gradually spontaneously recovers [46].

In the included studies, patients were mostly in their 4th-5th month of disease. Thus, during the middle follow-up of treatment, these patients were in the freezing period (4–12 months) of their disease course, when pain and dysfunction symptoms are most evident and treatment is most needed. So the PRP group is significantly more effective than the control group. While after 6 months or even a year of treatment, the patients have mostly entered the thawing period (12–42 months), and the natural course of the disease may also demonstrate reduced pain and gradual restoration of joint flexibility, and the PRP group may have a less obvious advantage compared to it. For example, Jeyaraman et al. [38] pointed out that in terms of grading the final treatment effect, with the effective rate of 87.0% in the PRP group compared with 84.4% in the control group, the difference between the two groups was not statistically significant, which may be related to the fact that the time to evaluate the efficacy is 1 year after treatment, at which time some patients may have entered the thawing period and may have natural remission.

In terms of safety, at present, most clinicians routinely choose oral or intra-articular injections of hormones and physiotherapy to treat FS [4, 58], with block therapy being the most common and most effective, however, there are numerous clinical and animal studies showing that the use of CS may cause cartilage damage [7] and even osteonecrosis [8], and the more the dose administered, the more significant the damaging effect. Therefore, CS should be avoided in the clinical treatment of joint pain. In contrast, in the included literature, only Jeyaraman et al. [38] reported that pain occurred in 17 patients (36.95%) and swelling in 7 patients (15.21%) after PRP treatment, while pain occurred in 23 patients in the control group (51.11%), indicating that there was no significant difference in the incidence of adverse effects between the two groups. In the remaining studies, except for Shahzad et al. [49] and Karabas et al. [47], which did not report adverse effects to treatment, other studies have reported no adverse effects, which may be related to the fact that PRP is an autologous blood component and there is no concern for rejection. Past studies on PRP also support that it is a safe and effective biologic therapy, with a post-treatment efficiency rate of about 70% after imaging assessment, significantly higher than that of about 40% in the control group [59], and a significantly lower recurrence rate than in the control group [60]. A study by Wang Heng et al. [61] noted that the complication rate after PRP use was 11.6% compared with 27.6% in the control group. Chen Juan et al. [62] noted that there was no significant difference in the rate of adverse effects between the PRP and control groups, both of which were mainly painful knee swelling, a common postoperative complication, and most of the symptoms disappeared within 6 h of treatment, this is highly consistent with the research results of Jeyaraman et al. [38]. Most of the adverse effects and complications reported in most studies were not related to PRP itself [63], so PRP has an advantage in terms of safety of treatment.

Overall, PRP was was identified as significantly relieve clinical symptoms, and have a higher efficiency rate and certain safety guarantees compared to other control treatment measures, supporting the hypothesis of this study.

### Advantages and limitations

Currently, several meta-analysis studies on relevant topics have been published, but the analysis of outcome indicators and the inclusion of relevant literature are not comprehensive, and there are cases in which some RCTs published within a corresponding period are not included in the analysis or RCTs are mixed with Cohort studies. This study included RCTs comprehensively and accurately, including a total of 14 RCTs. This will be the first meta-analysis in the world to comprehensively and in detail analyze the effect and safety of PRP for FS based only on existing RCTs, so as to provide some guidance for clinical treatment. This is the advantage and novelty of this study.

By the time of submission, only three relevant meta-analyses have been published [64–66], compared with the study of Nudelman et al. [65] and Yu et al. [66], this study included more original literature, there were 14 RCTs included in this study, and the outcome indicators included in this analysis were more comprehensive and detailed. In contrast, these two meta-analysis articles only included 4–5 original studies, including several cohort studies, and they included fewer outcome measures in the analysis. Compared with this study, Lin HW et al. [64] included 13 original articles, which are roughly the same as this study, but the items analyzed in his study were incomplete, and only active flexion, abduction, external rotation, and passive ones were included, no other direction. In addition, the UCLA, DASH, and SPADI were mixed together to meta-analyses, which may have some bias errors. The indicators included in this study are more comprehensive, and the analysis is more detailed and in-depth. This study only conducted descriptive analysis of non-RCTs and did not incorporate a data analysis, as previous research has indicated that these types of studies can influence the outcomes related to pain and ROM [64]. This is the advantage of this meta-analysis.

However, the present study has several limitations. First, there is no consensus in the current clinical studies on the preparation and use of PRP, which may cause some bias, as Supplemental Table 3, this limitation also exists in many meta-analyses on PRP in the past [67, 68], so this study adopts a conservative random effects model for analysis to reduce bias. Secondly, the sample sizes included were generally small, and more high-quality RCTs are needed to confirm the findings of this study. Thirdly, the variation in follow-up duration and the inconsistent recording of outcome indicators across different studies have influenced the assessment results and introduced potential bias. However, this paper has successfully minimized the impact of these factors. These factors may impact the level of evidence for the results.

## Conclusion

In conclusion, PRP therapy not only can relieve pain and functional impairment in FS patients in the short term compared to other therapy commonly used, but also can adequate sufficient safety and satisfactory consequences in medium to long-term follow-up. However, due to the small sample size of the study, the above conclusions need to be verified by more large samples, longer follow-up time and multicentre RCTs to better guide clinical decision-making.

## Electronic supplementary material

Below is the link to the electronic supplementary material.

Supplementary Material 1

Supplementary Material 2

Supplementary Material 3

Supplementary Material 4

## Data Availability

All data and materials are contained within the manuscript and its additional files.

## Abbreviations

FS: Frozen shoulder
PRP: Platelet-rich plasma
PRISMA: The Preferred Reporting Items for Systematic Reviews and Meta-Analyses
VAS: Visual analog score
ROM: Range of Motion
UCLA: The University of California at Los Angeles shoulder rating scale
SPADI: Shoulder Pain Disability Index
DASH: Disability of the Arm, Shoulder Hand Questionnaire
RCTs: Randomized controlled trials
non-RCT: Non-randomized controlled trial
RevMan 5.4: Review Manager 5.4
RR: Relative risk
CI: Confidence interval
MD: Mean difference
AICS: Articular injection corticosteroids
CS: Corticosteroids
Mean ± SD: Mean ± standard deviation
Median(IQR): Median(Interquartile range)
