# Supplementary material for: The clinical efficacy and safety of platelet-rich plasma on frozen shoulder: a systematic review and meta-analysis of randomized controlled trials
Source: BMC Musculoskelet Disord. 2024 Sep 6;25:718. doi: 10.1186/s12891-024-07629-1 (PMC11378644; doi:10.1186/s12891-024-07629-1)
Supplement: Supplementary file 3 — Supplementary Material 3 [file 12891_2024_7629_MOESM3_ESM.pdf]

Supplemental Appendix 1 Search Strategy

PubMed as an example:

1# Periarthritis[MeSH Terms]
2# bursitides[MeSH Terms]
3# Periarthritides
4# Frozen Shoulder
5# Shoulder, Frozen
6# Adhesive Capsulitis of the Shoulder
7# Shoulder Adhesive Capsulitis
8# Adhesive Capsulitides, Shoulder
9# Adhesive Capsulitis, Shoulder
10# Capsulitides, Shoulder Adhesive
11# Capsulitis, Shoulder Adhesive
12# Shoulder Adhesive Capsulitides
13# Capsulitis
14# Capsulitides
15# Pes Anserine Bursitis
16# Bursitides, Pes Anserine
17# Bursitis, Pes Anserine
18# Pes Anserine Bursitides
19# Adhesive Capsulitis
20# Adhesive Capsulitides
21# Capsulitides, Adhesive
22# Capsulitis, Adhesive
23# 1# OR 2# OR 3# OR 4# OR 5# OR 6# OR 7# OR 8# OR 9# OR 10# OR 11# OR 12# OR 13# OR 14# OR 15# OR 16# OR 17# OR 18# OR 19# OR 20# OR 21# OR 22#
24# Platelet-Rich Plasma[MeSH Terms]
25# Plasma, Platelet-Rich
26# Platelet Rich Plasma
27# Platelet-Rich Fibrin[MeSH Terms]
28# Fibrin, Platelet-Rich
29# Platelet Rich Fibrin
30# Leukocyte and Platelet-Rich Fibrin
31# L-PRF
32# Leukocyte and Platelet Rich Fibrin
33# PRP
34# PRF
35# 24# OR 25# OR 26# OR 27# OR 28# OR 29# OR 30# OR 31# OR 32# OR 33# OR 34#
36# 35# AND 23#

Web of science as an example:

1# ((((((((((ALL=(Platelet-Rich Fibrin)) OR ALL=(Platelet-Rich Plasma)) OR ALL=(Plasma, Platelet-Rich)) OR ALL=(PRP)) OR ALL=(PRF)) OR ALL=(Leukocyte and Platelet Rich Fibrin)) OR ALL=(L-PRF)) OR ALL=(Leukocyte and Platelet-Rich Fibrin)) OR ALL=(Platelet Rich Fibrin)) OR ALL=(Fibrin, Platelet-Rich)) OR ALL=(Platelet Rich Plasma)

2# (((((((((((((((((((((ALL=(Periarthritis)) OR ALL=(bursitides)) OR ALL=(Periarthritides)) OR ALL=(Frozen Shoulder)) OR ALL=(Shoulder, Frozen)) OR ALL=(Adhesive Capsulitis of the Shoulder)) OR ALL=(Shoulder Adhesive Capsulitis)) OR ALL=(Adhesive Capsulitides, Shoulder)) OR ALL=(Adhesive Capsulitis, Shoulder)) OR ALL=(Capsulitides, Shoulder Adhesive)) OR ALL=(Capsulitis, Shoulder Adhesive)) OR ALL=(Shoulder Adhesive Capsulitides)) OR ALL=(Capsulitis)) OR ALL=(Capsulitides)) OR ALL=(Pes Anserine Bursitis)) OR ALL=(Bursitides, Pes Anserine)) OR ALL=(Bursitis, Pes Anserine)) OR ALL=(Pes Anserine Bursitides)) OR ALL=(Adhesive Capsulitis)) OR ALL=(Adhesive Capsulitides)) OR ALL=(Capsulitides, Adhesive)) OR ALL=(Capsulitis, Adhesive)

3# 1# AND 2#
